# Profiling tissue-resident T cell repertoires by RNA sequencing

**DOI:** 10.1186/s13073-015-0248-x

**Published:** 2015-11-30

**Authors:** Scott D. Brown, Lisa A. Raeburn, Robert A. Holt

**Affiliations:** Canada’s Michael Smith Genome Sciences Centre, BC Cancer Agency, Vancouver, British Columbia V5Z 1L3 Canada; Genome Science and Technology Program, University of British Columbia, Vancouver, British Columbia V6T 1Z4 Canada; Department of Molecular Biology and Biochemistry, Simon Fraser University, Burnaby, British Columbia V5A 1S6 Canada; Department of Medical Genetics, University of British Columbia, Vancouver, British Columbia V6T 1Z4 Canada

## Abstract

Deep sequencing of recombined T cell receptor (TCR) genes and transcripts has provided a view of T cell repertoire diversity at an unprecedented resolution. Beyond profiling peripheral blood, analysis of tissue-resident T cells provides further insight into immune-related diseases. We describe the extraction of TCR sequence information directly from RNA-sequencing data from 6738 tumor and 604 control tissues, with a typical yield of 1 TCR per 10 million reads. This method circumvents the need for PCR amplification of the TCR template and provides TCR information in the context of global gene expression, allowing integrated analysis of extensive RNA-sequencing data resources.

**Electronic supplementary material:**

The online version of this article (doi:10.1186/s13073-015-0248-x) contains supplementary material, which is available to authorized users.

## Background

Primary sequence analysis of the highly variable complementarity determining region 3 (CDR3) of rearranged T cell receptor (TCR) genes provides insight into the adaptive immune response. T cells recognize peptide epitopes presented on the surface of cells on major histocompatibility complex (MHC) molecules. CDR3 is the TCR motif that directly binds MHC-presented peptide epitopes and this binding interaction is the main factor conferring T cell antigen specificity. Typically, CDR3 sequence information is acquired by performing TCR-sequencing (TCR-seq) experiments on peripheral T cells isolated from blood [1, 2]; amplifying the CDR3 region with a conserved C gene primer followed by 5′ rapid amplification of cDNA ends [2], or with a multiplexed set of V and J gene primers [3]. TCR-seq applied to tissue specimens can provide insight into tumor-infiltrating lymphocytes [4, 5], T cells associated with autoimmune pathology [6–8] and infection [9], and the properties of normal primary and secondary lymphatic tissues [10, 11].

**Fig. 1:**
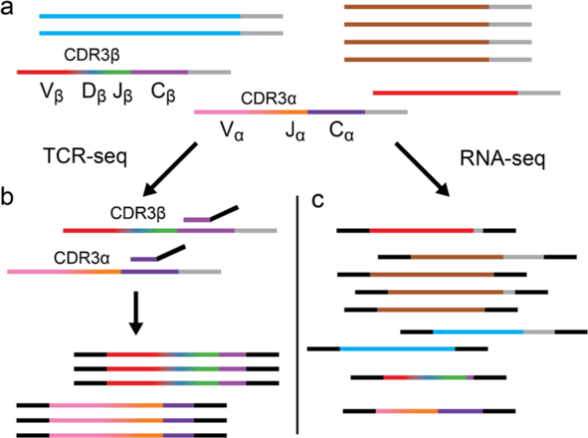
Schematic representation of T cell receptor sequencing (*TCR-seq*) versus RNA sequencing. *Horizontal lines* represent mRNA transcripts with gray poly-A tails. Each color represents a unique gene sequence. **a** A pool of all mRNA in a sample is depicted, which contains irrelevant transcripts (*blue*, *brown*, and *red*) as well as recombined TCR transcripts (*multi-colored*). **b** TCR-seq involves selective amplification of the CDR3 region of TCR transcripts (displayed as a color gradient) by reverse transcription polymerase chain reaction (PCR) shown using a conserved C-gene primer (*purple* with black sequencing adapter tails) for the initial reverse transcription step and resulting, after PCR (not shown), in an enriched set of recombined TCR sequences. **c** RNA-seq employs shotgun sequencing, generating fragments from all transcripts present in the sample, which then have sequencing adapters ligated (*black*). The resulting sequencing library will contain fragments that, by chance, contain the CDR3 encoding sequence. Additionally, these libraries may contain fragments that share sequence similarity to recombined TCR sequences (e.g., the red transcript), potentially leading to false-positives

Conventional TCR-seq methods provide a detailed view of TCR diversity [2, 3, 12]. However, because they rely on targeted amplicon sequencing, they do not evaluate TCR variation in the context of the overall genetic diversity of the specimen from which the data are derived. Next-generation sequencing technology has made whole-genome and transcriptome sequencing routine, and provided opportunities for the extraction of immunological data, such as human leukocyte antigen (HLA) type, using specialized software tools [13, 14]. Here, we describe an optimized approach for TCR CDR3 extraction from RNA-seq datasets from solid tumors, for the purpose of characterizing T cell populations present in the tumor environment. Compared to TCR-seq, the main challenge in CDR3 extraction from tumor RNA-seq data is the disproportionally large number of non-TCR transcripts (Fig. 1). For a pure lymphocyte population, only one in approximately 2000 transcripts are TCR transcripts (see “Methods”) and in tissues T cells represent a minor cell type, further decreasing TCR transcript representation. This necessitates an analytical approach that is both fast and accurate for TCR extraction from tissue-derived RNA-seq datasets.

## Methods

### Ethics

The research described herein conformed to the Helsinki Declaration. All clinical specimens not part of The Cancer Genome Atlas (TCGA) were obtained previously [15] with informed consent by the British Columbia Cancer Agency Tumor Tissue Repository, which operates as a dedicated biobank with approval from the University of British Columbia-British Columbia Cancer Agency Research Ethics Board (certificate #H09-01268).

### Extraction of T cell receptor CDR3 sequences from RNA-seq data

We deployed MiTCR [16] v1.0.3, which is well suited for the annotation of CDR3 sequences from sequencing reads. However, upon initial application of MiTCR to tumor RNA-seq data using the default parameters, we identified hundreds of non-specific and out-of-frame CDR3 sequences per sample, which prompted us to explore alternative parameters. Closer inspection of the bogus CDR3s identified low similarity between these sequences and the putative flanking TCR V and J gene segments, suggesting that the false positives were spurious, non-TCR hits to TCR-like sequences elsewhere in the transcriptome. Therefore, we optimized settings using positive and negative control RNA-seq data. The positive control dataset comprised TCR sequences generated in silico, as follows: V, (D), J, and C gene reference sequences for human TCR alpha and beta chains were downloaded from the ImMunoGeneTics information system [17]. To generate each transcript, V, (D), J, and C genes were chosen randomly. Non-templated nucleotide addition and deletion frequencies at the V-(D)-J gene junctions were modeled from observed frequencies in normal TCR chain beta repertoires [2]. Owing to the absence of D genes in the alpha chain, the number of bases added between the V and J genes was selected by averaging the number to add to both the V-D and D-J junctions in a beta chain. Out-of-frame transcripts and those that contained stop codons were removed. Full-length recombined TCR transcript sequences were run through MiTCR using stringent alignment parameters (minimum V and J alignment length both set to 20 in the XML parameter file; default value is 12) to annotate the CDR3 region in the transcript, and to ensure the in silico recombination created a CDR3 sequence able to be detected by MiTCR. We generated 10,000 transcripts each of alpha and beta, with 8573 alpha and 8804 beta sequences successfully being identified by MiTCR and being used as the source for the positive control dataset. The distribution of CDR3 lengths for the in silico generated alpha and beta chains are displayed in Additional file 1: Figure S1.

For negative control RNA-seq data, we used paired-end 101-nucleotide RNA-seq data from seven TCR-negative cell lines, downloaded from ENCODE [18] and pooled for use as a negative control (Additional file 1: Table S1)*.* To create negative datasets for shorter read lengths, reads from the 101-nucleotide datasets were truncated to 76-nucleotide and 50-nucleotide reads. For positive control datasets, error-free reads (101, 76, and 50 nucleotides) were created for each in silico generated CDR3, with the center of the CDR3 region positioned at the center of the read.

An unbiased parameter space exploration was performed across all pairwise combinations of V gene minimum alignments and J gene minimum alignments (values 8 to 26 explored, all other parameters set as default) to determine optimal parameters. For each of the 361 parameter pairs, MiTCR was run on the negative and positive control datasets. For negative control datasets, the number of detected bogus CDR3s was tracked, and for positive control datasets, the number of correctly annotated CDR3s was tracked. Optimal parameters were assessed for each TCR chain–read length combination. Sensitivity was calculated for each parameter pair by dividing the number of recovered CDR3s by the maximum number of recovered CDR3s for all parameter pairs of that TCR chain–read length combination, giving a relative sensitivity value. We used a binary categorization to bin the false discovery rates as acceptable or not. For a set of false discovery rates, we selected the best parameter pair that had an acceptable false discovery rate and highest sensitivity. In the case of multiple parameter pairs being equally acceptable, the pair that minimized the V and J alignment parameters was selected. These optimal parameters are summarized in Additional file 1: Table S2.

### Benchmarking CDR3 extraction efficiency using simulated data

The Flux Simulator [19] v1.2.1 is a computational tool that generates RNA-seq datasets by simulating a transcriptome expression profile, library construction, and sequencing errors. We simulated a range of sequencing depths (10^4^–10^8^ reads) and read lengths (50, 76, and 101 nucleotides) to determine the importance of different factors on characterizing the ability to detect a given CDR3 sequence. Full-length in silico recombined TCR sequences were annotated as single exon genes in a reference synthetic chromosome sequence file, which we added to the human genome (GRCh38) to be used as the reference genome for Flux Simulator. Flux Simulator was run with the following command line flags: −t simulator, −x (to simulate expression), −l (to simulate library construction), −s (to simulate sequencing), and –p parameterFile.par. The parameter file contained the following parameters: REF_FILE_NAME: path to .gtf file; GEN_DIR: directory with genome reference files; FASTA: true; ERR_FILE: 76; READ_LENGTH: one of 50, 76, 101; PAIRED_END: true, UNIQUE_IDS: true; READ_NUMBER: one of 10000, 50000, 100000, 500000, 1000000, 5000000, 10000000, 50000000, 100000000; and TMP_DIR: path to temporary directory.

Ten RNA-seq datasets were simulated for each read length and sequencing depth combination to minimize the risk of any stochastic effects on transcript abundance in any one simulation confounding the variables that explain CDR3 recovery. Each simulated dataset was run through MiTCR using the optimized parameter sets for a theoretical 0 % false discovery rate, and results from all 270 simulations were pooled for analysis (Additional file 1: Figure S2). There are two requirements for detection of a CDR3: (1) the TCR transcript must be expressed, and (2) the sequence read length must be longer than the CDR3 length (Additional file 1: Figure S3). Before modeling, we subsetted the data to cases that met these two criteria (n = 362,233). A multivariate logistic regression model was fit using half of the data (n = 181,116), leaving half the data for a validation set (n = 181,117). The fit of the logit function is shown in Equ. 1. The performance of the model is summarized in Additional file 1: Table S3, and resulted in 63.8 % sensitivity and 93.1 % specificity.

1 $$ \begin{array}{l} logit\left(CDR3\  detected\right)=\\ {}-5.38+1.98\left({ \log}_{10}\left( transcripts\  per\  million\right)\right)+0.51\left(\frac{sequencing\  depth}{10,000,000}\right)\\ {}+0.04\left( read\  length\right)-0.04\left(CDR3\  length\right)\end{array} $$

### Approximation of TCR transcript abundance from percent T cell infiltration

We queried Illumina BodyMap (http://www.ebi.ac.uk/gxa/experiments/E-MTAB-513) and GTEx (http://www.gtexportal.org/home/) to find the expression of *TRAC* and *TRBC1/2* in healthy whole blood. An average expression of approximately 150 fragments per kilobase of transcript per million mapped reads was observed for these genes. Because these genes are roughly 1 kb in length, this level of expression translates to a transcript fraction on the order of 1.5 × 10^−4^. Because lymphocytes are generally between 20 and 40 % of white blood cells, a pure lymphocyte population would have a TCR transcript fraction of approximately 5 × 10^−4^. Assuming a similar cellular composition between peripheral blood lymphocytes and tumor infiltrating lymphocytes (TIL), a tumor with 2 % TIL (the median value for TCGA tumors, parsed from TCGA biospecimen slide data) would have a TCR transcript fraction of 1 × 10^−5^. This value can be inserted into the logistic regression model in order to predict the minimum sequence depth required to have a 50 % chance of detecting a CDR3 sequence with this abundance and other known properties.

### TCGA RNA-seq data analysis

All available RNA-seq fastq files for solid tumors and matched normal tissues were downloaded with permission from Cancer Genomics Hub (https://cghub.ucsc.edu/). This included 8655 samples from 24 tumor sites. The majority (79 %) of this TCGA RNA-seq data are 50-nucleotide reads, while 21 % are 76 nucleotides, with sequencing depths ranging from 5.27 × 10^6^ to 4.51 × 10^8^ reads. To be able to directly compare the extracted CDR3s across all samples, we performed a pre-normalization step by truncating all reads to 50 nucleotides, and randomly sub-sampling 1.00 × 10^8^ reads from every sample. This resulted in removal of 41.5 % of all sequence data (5.20 × 10^11^ reads) and 15.2 % of samples (1313) owing to insufficient depth, leaving 7342 (6738 tumor and 604 normal) samples and 7.34 × 10^11^ total reads for analysis. Prior to running MiTCR, the fastq files were cleaned by only retaining reads longer than 40 nucleotides and reads containing standard (ACTGN) bases. For every sample, MiTCR was run with the optimized V and J alignment parameters for a theoretical 0 % false discovery rate with 50 nucleotide reads for both alpha and beta chains, keeping all other parameters default. Extracted CDR3 sequences that contained stop codons or frame shifts were removed prior to all further analysis.

### TCGA gene expression datasets

In order to correlate extracted TCR diversity with the expression of immune-related genes, all available RNASeqV2 data from the TCGA Data Portal were downloaded. These data provide gene expression information generated using MapSplice [20] for alignment and RSEM [21] to quantify gene expression. The reported *scaled_estimate* value was multiplied by 10^6^ to obtain transcripts per million (TPM). To obtain consensus gene expression values, we summed the TPM values within each of the following groups: HLA Class I (*HLA-A*, *HLA-B*, *HLA-C*, *HLA-E*, *HLA-F*, *HLA-G*), Class II (*HLA-DMA*, *HLA-DMB*, *HLA-DOA*, *HLA-DOB*, *HLA-DPA1*, *HLA-DPB1*, *HLA-DQA1*, *HLA-DQA2*, *HLA-DQB1*, *HLA-DQB2*, *HLA-DRA*, *HLA-DRB1*, *HLA-DRB5*), CD8 (*CD8A*, *CD8B*), or CD3 (*CD3D*, *CD3E*, *CD3G*). Pearson correlations were calculated between these genes and the number of distinct CDR3 sequences in each individual (Additional file 1: Figures S4 and S5).

### Inferred pairing of TCR alpha and beta subunits

For each tumor sample, all possible pairwise combinations of TCR alpha and beta subunit CDR3 sequences derived from that sample were specified (n = 1,286,810). We then looked for recurrent alpha-beta pairs among all TCGA tumor samples, and identified 188 distinct alpha-beta pairs that were found in at least two individuals. To test if this was a stronger alpha-beta co-occurrence than would be expected by chance, we randomized the relationship between sample identifiers and their corresponding TCR alpha and beta sequences. We then regenerated all possible pairwise combinations within subjects and determined the frequency of recurrent alpha-beta pairs in this randomized dataset. Randomization was repeated for 100 iterations, and the proportion of trials that had a degree of sharing greater than or equal to the original, non-randomized data was taken as the *P* value.

### Shared peptide-MHC and CDR3 sequences

We predicted HLA Class I alleles and MHC-presented point mutations for 1361 TCGA individuals as previously described [22], with an IC50 value of 500 nM for MHC-predicted binding affinity taken as the maximum threshold for potential immunogenicity. We counted the frequency of each peptide-MHC (pMHC; n = 305,438), and found 393 to be recurrent. In order to determine if any of the individuals sharing a pMHC also shared a similar CDR3 sequence, we took all CDR3 sequences from all 1361 individuals, and clustered them to allow for inexact CDR3 sequence matches. We clustered at 95 % identity using CD-HIT [23, 24] v4.6 with the following parameters: −c 0.95; −n 5; −l 5. We then checked each cluster to see if it contained sequences from at least two individuals, and if those individuals shared a pMHC. We found one cluster of two individuals that met this criteria. To test how frequently this would be expected to happen by chance, we randomly selected two individuals from the set, and checked if they shared a pMHC. We measured the fraction of successes from 1,000,000 trials, then multiplied by the number of clusters that contained two individuals (n = 1457) to correct for multiple testing to give the adjusted *P* value.

### Data analysis

All data analysis was performed in R [25] v3.1.2 or Python (http://www.python.org) v2.4.3 and v3.2.2.

### Code availability

Custom code is available upon request.

## Results and discussion

### Somatically rearranged T cell receptor sequences can be effectively recovered from RNA-seq data

We optimized the extraction of TCR alpha and beta chain sequences from RNA-seq datasets by evaluating negative and positive control datasets and adjusting search parameters. We identified optimal V and J alignment parameters that yielded an average of 94 % sensitivity for 100 % specificity using the shortest (50 nucleotide) reads (Additional file 1: Table S2, see “Methods”). Sensitivity was limited, ultimately, by the inability to detect the small proportion of CDR3s that are longer than a sequencing read (Additional file 1: Figure S3).

To estimate the yield of TCR transcripts that could be expected from typical RNA-seq experiments, we used Flux Simulator [19] to generate simulated RNA-seq data spiked with in silico recombined TCR transcripts, and processed these data as described in “Methods”. As expected, the most abundant TCR transcripts were the most readily detected and the sensitivity of the method increased with increasing sequencing depths and read lengths (Additional file 1: Figure S2). We fit a multivariate logistic regression model to explain the odds of detecting a CDR3 sequence from the RNA-seq data using the explanatory variables log_10_(transcripts per million) (odds ratio [OR] 7.242; 95 % confidence interval [CI] 7.079, 7.411; *P* < 2 × 10^−16^), sequencing depth in tens of millions of reads (OR 1.667; 95 % CI 1.658, 1.677; *P* < 2 × 10^−16^), sequence read length (OR 1.0358; 95 % CI 1.035, 1.037; *P* < 2 × 10^−16^), and CDR3 nucleotide sequence length (OR 0.958; 95 % CI 0.956, 0.960; *P* < 2 × 10^−16^), and observed that initial TCR transcript abundance is the most important factor in predicting whether a CDR3 would be detected.

For tumor tissue, where the degree of T cell infiltration is typically about 2 % (see “Methods”), we would expect 0.001 % of the total transcripts to be recombined TCR transcripts. Assuming a monoclonal infiltrate, an RNA-seq depth of 70 million 50-nucleotide reads is required to have a greater than 50 % chance of detecting a 45-nucleotide CDR3 (the most frequent CDR3β length [2]). Additional probabilities are presented in Additional file 1: Table S4. To further evaluate the yield of TCR sequences from RNA-seq data, we generated TCR-seq for TCR beta chain (1,411,056 reads; 2823 unique CDR3β sequences) and RNA-seq for total cDNA (56,067,687 reads; 9 unique CDR3β sequences) data from the same colorectal tumor tissue sample, using previously described methods [12, 26]. We observed that all high confidence CDR3βs identified by RNA-seq (n = 9) fell within the top 2.1 % (n = 60) of CDR3βs detected by TCR-seq, ranked by abundance (data not shown), confirming that at a modest depth of sequencing, RNA-seq can identify the most abundant CDR3-encoding transcripts. The most abundant T cells may or may not be the most biologically relevant T cells. In solid tumors, the relationship between clonal abundance of T cells and anti-tumor immunity is not yet clear—it is obscured by the presence of bystander T cells in the tumor environment as further discussed below. In cases such as T cell leukemia, where the T cell is the cancerous cell and is highly expanded, the most abundant CDR3 sequences found by RNA-seq should generally be adequate to identify the tumor clone and monitor disease progression [27]. Likewise, in cases such as acute infection, the most abundant T cells are likely those that are most biologically relevant. TCR transcripts from rare T cells will become more accessible in future because continually declining sequencing costs will allow deeper and deeper transcriptome sampling by RNA-seq.

### TCR sequence diversity in tumor-associated T cell repertoires

**Fig. 2:**
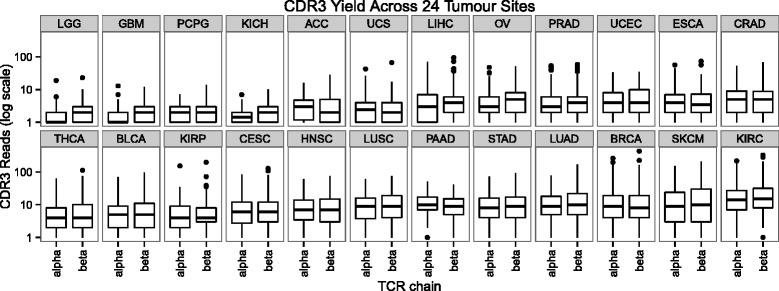
The number of reads containing CDR3 sequences varies across tumor sites. *ACC* adrenocortical carcinoma, *BLCA* bladder urothelial carcinoma, *BRCA* breast invasive carcinoma, *CESC* cervical squamous cell carcinoma and endocervical adenocarcinoma, *CRAD* colon and rectum adenocarcinoma, *ESCA* esophageal carcinoma, *GBM* glioblastoma multiforme, *HNSC* head and neck squamous cell carcinoma, *KICH* kidney chromophobe, *KIRC* kidney renal clear cell carcinoma, *KIRP* kidney renal papillary cell carcinoma, *LGG* brain lower grade glioma, *LIHC* liver hepatocellular carcinoma, *LUAD* lung adenocarcinoma, *LUSC* lung squamous cell carcinoma, *OV* ovarian serous cystadenocarcinoma, *PAAD* pancreatic adenocarcinoma, *PCPG* pheochromocytoma and paraganglioma, *PRAD* prostate adenocarcinoma, *SKCM* skin cutaneous melanoma, *STAD* stomach adenocarcinoma, *TCR* T cell receptor, *THCA* thyroid carcinoma, *UCEC* uterine corpus endometrial carcinoma, *UCS* uterine carcinosarcoma

**Fig. 3:**
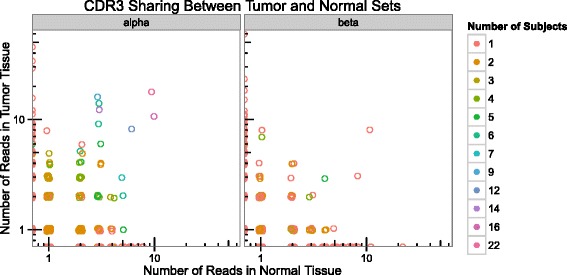
The majority of CDR3s recovered from tumor/normal control tissue pairs are unique to tumor or normal tissue. For every CDR3, the number of reads in the set of tumors is plotted on the y-axis, with the number of reads in the set of normal samples on the x-axis. *Circles* are colored by the number of individuals in which that CDR3 sequence is detected

We extracted TCR alpha and beta chain CDR3 sequences from all available RNA-seq datasets from the TCGA project. This included 7342 total datasets derived from 6738 solid tumor and 604 matched normal tissues, from 24 different tumor sites. In tumors, the yield per individual ranged from 0 to 702 (median of 9) reads containing a full CDR3 sequence (Fig. 2), and this translated to a range of 0 to 538 (median of 7) distinct CDR3 amino acid sequences per individual. Kidney renal clear cell carcinoma (KIRC) produced the greatest yield of CDR3s, whereas brain lower grade glioma produced the lowest. As expected, there was a strong correlation between number of distinct CDR3 amino acid sequences and *CD3* expression (Additional file 1: Figure S4). Comparing the gene expression of HLA Class I and Class II genes with the number of distinct CDR3 amino acid sequences per individual, we observed a positive correlation, with markedly stronger correlations seen for Class II genes (Additional file 1: Figure S5; *P* = 9.3 × 10^−10^, paired *t* test). This is consistent with recent reports highlighting the immunoreactivity of T cells with specificity for MHC Class II presented tumor antigens [28–30]. Next, we evaluated the differential abundance of CDR3s between tumors and matched normal control tissues for all individuals where the RNA-seq data were available for both (n = 462; Fig. 3 and Additional file 1: Table S5). Of 6611 total alpha chains in this set, 3560 (53.8 %) were unique to tumor samples and 2826 (42.7 %) were unique to matched control samples. Likewise, of the 7664 beta chains, 4279 (55.8 %) were unique to tumor and 3277 (42.8 %) were unique to matched control samples. A total of 225 unique CDR3α and 108 unique CDR3β sequences were present in both tumor and control tissues. Thus, while there is evidence for a larger and more diverse T cell infiltrate in tumor compared to control tissues (*P* < 2.2 × 10^−16^, chi-squared test), these results suggest that a large proportion of tumor-associated T cells are bystanders, not readily distinguishable from the normal population of tissue resident T cells. A single individual with KIRC was a notable outlier in this analysis. The tumor sample from this individual yielded the three most abundant tumor-specific CDR3αs and the two most abundant tumor-specific CDR3βs in the entire cohort, suggesting the possibility of an acute anti-tumor T cell response in this individual.

### Public T cells are common in the tumor environment

**Fig. 4:**
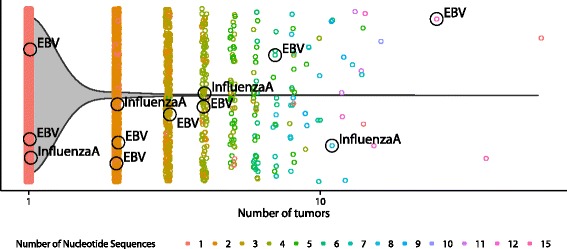
Sharing of CDR3β sequences. All 49,672 CDR3β sequences derived from tumors are plotted along the x-axis according to the number of tumors they are found in. *Color* defines the number of nucleotide sequences that were found to generate the same CDR3β amino acid sequence. The violin plot overlay shows that most recovered CDR3β sequences are unique to an individual, though there is notable sharing (4.4 %) between individuals. Known public viral-specific CDR3β sequences [31] are labeled with their antigen specificity. *EBV* Epstein–Barr virus

To explore the recurrence of TCRs mined from the tumor environment, we compared the CDR3β sequences extracted from the complete set of analyzed TCGA samples to the approximately 1.1 million distinct CDR3β sequences we previously identified by deep TCR-seq analysis of peripheral blood from a healthy volunteer [12]. Of all 49,672 distinct TCGA CDR3β sequences we observed, 22.8 % were found in the peripheral repertoire of the healthy individual (Additional file 1: Figure S6). We found the level of overlap for those CDR3βs that were seen in multiple TCGA individuals (76.5 % of 2197 shared TCGA CDR3βs found in the healthy repertoire) was substantially greater than for those that were unique to a single TCGA individual (20.3 % of 47,475 unique TCGA CDR3βs found in the healthy repertoire), suggesting these shared tumor-associated CDR3βs are derived, predominantly, from public T cells. Indeed, when we queried tumor-associated CDR3β sequences for matches to CDR3β sequences in the literature with defined antigen specificity, we found numerous matches to known viral-specific TCRs [31] (Fig. 4). TCGA CDR3βs with viral specificity were much more common within the set of shared TCGA CDR3βs than within the set of unique TCGA CDR3βs. Specifically, nine of 2197 CDR3βs (0.41 %) that were shared among TCGA individuals were identifiable as being viral-specific, whereas only three of the 47,475 CDR3βs (0.0063 %) that were unique to a single TCGA individual were similarly identifiable.

Given the observation of substantial sharing of CDR3 sequences among individuals, we asked if some of the shared alpha and beta CDR3 sequences may represent shared dimeric TCRs. To test this we generated all possible alpha-beta pairs within each individual’s alpha and beta repertoires, and looked for sharing of any pairs between two or more individuals. We observed 188 distinct alpha-beta pairs that were found in at least two individuals, which was not significantly more than would be expected by chance (*P* = 0.42, random resampling, see “Methods”). We also asked if individuals with shared mutations and shared HLA alleles may also share TCR sequences. Previously, for a subset of TCGA individuals, we identified tumor point mutations predicted to yield MHC class I binding peptides (pMHCs) [22]. We clustered the CDR3 amino acid sequences from all individuals with predicted mutant pMHCs at 95 % amino acid sequence identity. Of the 17,092 total CDR3 sequence clusters (including singletons), only one cluster contained sequences from two individuals with matching mutant pMHCs (Additional file 1: Table S6). Although this is not statistically significant (*P* = 0.35, random resampling, see “Methods”), with deeper sequencing data from large numbers of individuals, this approach may prove useful for matching TCR sequences to the neo-antigens they recognize.

## Conclusions

In future, as sequence costs continue to decline, there will be increasing opportunities to derive immune signatures from unbiased data types. Here, we have optimized an analytical strategy for extracting T cell repertoire information from RNA-seq datasets, and used it to characterize tumor-associated T cell repertoires. We have provided optimal parameters for mining RNA-seq datasets of varying read lengths, and provided a range of parameters for varying levels of acceptable false positive rates. This procedure was validated on simulated RNA-seq datasets with known recombined TCR transcripts, and was also compared to classical TCR-seq data, showing that the subset of TCRs we detected using RNA-seq were the most abundant T cells in the sample. The expected yield of TCR reads from RNA-seq data is ultimately dependent on the level of T cell infiltration in the sample and the clonality of the infiltrate. Assuming a similar cellular composition to TCGA tumors, one can expect on the order of one TCR read from 10 million sequence reads. Our analysis has highlighted a strong and novel correlation between tumor TCR diversity and tumor MHC Class II expression and high prevalence of public T cells in the tumor environment. Further, within the limitations of the available data, we have explored the association between alpha-beta TCR pairs, and linking TCR sequences to specific pMHC complexes. Analyses of this nature may inform future cancer immunotherapy strategies, and we expect that this same approach will have value in exploring other immune-related pathologies, where large RNA-seq datasets already exist or can be obtained.

## Additional file

Additional file 1:
**Figures S1–S6, and Tables S1–S6.** (DOCX 1784 kb)
