# Supplementary material for: Profiling tissue-resident T cell repertoires by RNA sequencing
Source: Genome Med. 2015 Nov 30;7:125. doi: 10.1186/s13073-015-0248-x (PMC4666197; doi:10.1186/s13073-015-0248-x)
Supplement: Additional file 1: — Figures S1–S6, and Tables S1–S6. (DOCX 1784 kb) [file 13073_2015_248_MOESM1_ESM.docx]

**SUPPLEMENTARY DATA**

**Supplementary Table 1.** Table of ENCODE datasets which were merged for use as a negative control.

| Library | Biosample | File Accessions | Total number of reads |
| --- | --- | --- | --- |
| ENCLB113QPT | ENCBS780PCJ - smooth muscle cell | ENCFF548JWS  ENCFF004IRQ | 208,920,126 |
| ENCLB615ALX | ENCBS077RUJ – hepatocyte | ENCFF245VTB  ENCFF369QXD | 200,577,302 |
| ENCLB160QNF | ENCBS018TPT - neural progenitor cell | ENCFF939FVE  ENCFF201WLO | 202,290,896 |
| ENCLB714MUL | ENCBS514GVM - SK-N-DZ | ENCFF482SFO  ENCFF691TRA | 156,710,634 |
| ENCLB534MTC | ENCBS234AAA - LHCN-M2 | ENCFF119TIN  ENCFF494PBN | 173,859,996 |
| ENCLB011AUM | ENCBS367AAA - fibroblast of arm | ENCFF002DMN  ENCFF002DMO | 182,860,148 |
| ENCLB059TNM | ENCBS518AAA - SK-MEL-5 | ENCFF002DLD  ENCFF002DLF | 196,017,866 |
| All Combined |  |  | 1,317,236,968 |

**Supplementary Table 2.** Table of optimized parameters for a range of false discovery rates.

|  |  |  |  | **minAlignmentMatches Parameter** | |
| --- | --- | --- | --- | --- | --- |
| **Allowed false positives per 100M reads** | **Sensitivity (%)^a^** | **TCR Chain** | **Read length** | **V** | **J** |
| 0 | 98.15 | Alpha | 50 | 10 | 20 |
| 0 | 90.76 | Beta | 50 | 12 | 16 |
| 0 | 100 | Alpha | 76 | 18 | 11 |
| 0 | 99.98 | Beta | 76 | 12 | 18 |
| 0 | 100 | Alpha | 101 | 12 | 19 |
| 0 | 100 | Beta | 101 | 14 | 16 |
| 1 | 98.55 | Alpha | 50 | 10 | 17 |
| 1 | 94.73 | Beta | 50 | 13 | 14 |
| 1 | 100 | Alpha | 76 | 17 | 11 |
| 1 | 99.99 | Beta | 76 | 8 | 18 |
| 1 | 100 | Alpha | 101 | 19 | 9 |
| 1 | 100 | Beta | 101 | 14 | 14 |
| 5 | 98.70 | Alpha | 50 | 8 | 17 |
| 5 | 97.00 | Beta | 50 | 12 | 13 |
| 5 | 100 | Alpha | 76 | 12 | 15 |
| 5 | 99.99 | Beta | 76 | 12 | 14 |
| 5 | 100 | Alpha | 101 | 14 | 14 |
| 5 | 100 | Beta | 101 | 8 | 17 |
| 10 | 98.89 | Alpha | 50 | 10 | 15 |
| 10 | 97.85 | Beta | 50 | 12 | 12 |
| 10 | 100 | Alpha | 76 | 10 | 16 |
| 10 | 99.99 | Beta | 76 | 11 | 14 |
| 10 | 100 | Alpha | 101 | 12 | 15 |
| 10 | 100 | Beta | 101 | 9 | 16 |

^a^ Calculated as the count of CDR3s recovered with that parameter pair divided by the maximum count of CDR3s recovered in all parameter pairs tested.

**Supplementary Table 3.** Observed and predicted detection of CDR3s in the validation set by logistic regression with cut-off of 0.50.

|  | Predicted | |  |
| --- | --- | --- | --- |
| Observed | **Detected** | **Not Detected** | **% Correct** |
| Detected | 31163 | 17698 | 63.8 |
| Not Detected | 9162 | 123094 | 93.1 |
| Overall |  |  | 85.2 |

**Supplementary Table 4.** Table of predictions from model for some relevant explanatory variable values. Underlined values vary within each group.

| Transcript Fraction | Sequencing Depth | Read Length | CDR3 Length | Probability of detection (95% CI) |
| --- | --- | --- | --- | --- |
| 1 × 10^-5^ | 70,000,000 | 50 | 45 | 0.503 (0.495 – 0.512) |
| 1 × 10^-6^ | 50,000,000 | 76 | 48 | 0.100 (0.097 – 0.102) |
| 5 × 10^-6^ | 50,000,000 | 76 | 48 | 0.306 (0.302 – 0.311) |
| 1 × 10^-5^ | 50,000,000 | 76 | 48 | 0.445 (0.440 – 0.450) |
| 2.5 × 10^-5^ | 50,000,000 | 76 | 48 | 0.638 (0.633 – 0.643) |
| 1 × 10^-5^ | 10,000,000 | 76 | 48 | 0.094 (0.092 – 0.096) |
| 1 × 10^-5^ | 25,000,000 | 76 | 48 | 0.183 (0.180 – 0.186) |
| 1 × 10^-5^ | 50,000,000 | 76 | 48 | 0.445 (0.440 – 0.450) |
| 1 × 10^-5^ | 100,000,000 | 76 | 48 | 0.912 (0.908 – 0.915) |
| 1 × 10^-5^ | 50,000,000 | 50 | 48 | 0.243 (0.237 – 0.249) |
| 1 × 10^-5^ | 50,000,000 | 76 | 48 | 0.445 (0.440 – 0.450) |
| 1 × 10^-5^ | 50,000,000 | 101 | 48 | 0.659 (0.653 – 0.665) |
| 1 × 10^-5^ | 50,000,000 | 50 | 41 | 0.302 (0.296 – 0.308) |
| 1 × 10^-5^ | 50,000,000 | 50 | 45 | 0.267 (0.261 – 0.273) |
| 1 × 10^-5^ | 50,000,000 | 50 | 48 | 0.243 (0.237 – 0.249) |
| 1 × 10^-5^ | 50,000,000 | 76 | 39 | 0.541 (0.535 – 0.546) |
| 1 × 10^-5^ | 50,000,000 | 76 | 45 | 0.477 (0.472 – 0.482) |
| 1 × 10^-5^ | 50,000,000 | 76 | 51 | 0.414 (0.408 – 0.420) |

**Supplementary Table 5.** Sample numbers for tumor-normal pairs in each tumor site.

| Tumor Site | Number of Samples |
| --- | --- |
| BRCA | 96 |
| KIRC | 56 |
| THCA | 47 |
| LUSC | 42 |
| PRAD | 40 |
| HNSC | 34 |
| STAD | 30 |
| LIHC | 27 |
| CRAD | 21 |
| KIRP | 15 |
| KICH | 14 |
| LUAD | 13 |
| ESCA | 10 |
| BLCA | 9 |
| CESC | 3 |
| UCEC | 3 |
| PCPG | 2 |
| Total | 462 |

**Supplementary Table 6.** Summary of a CDR3 sequence cluster that shares pMHC.

| Cluster Number | Subjects | Sequences | Mutant Gene | Peptide | HLA |
| --- | --- | --- | --- | --- | --- |
| 6437 | TCGA-HU-A4G8 | CASSRDSSYEQYF | PGM5 | GRLIIGQNGV | B*27:05P |
| 6437 | TCGA-BR-8081 | CASSLRDSSYEQYF | PGM5 | GRLIIGQNGV | B*27:05P |
| 6437 | TCGA-HU-A4G8 | CASSRDSSYEQYF | PGM5 | GRLIIGQNGVL | B*27:05P |
| 6437 | TCGA-BR-8081 | CASSLRDSSYEQYF | PGM5 | GRLIIGQNGVL | B*27:05P |


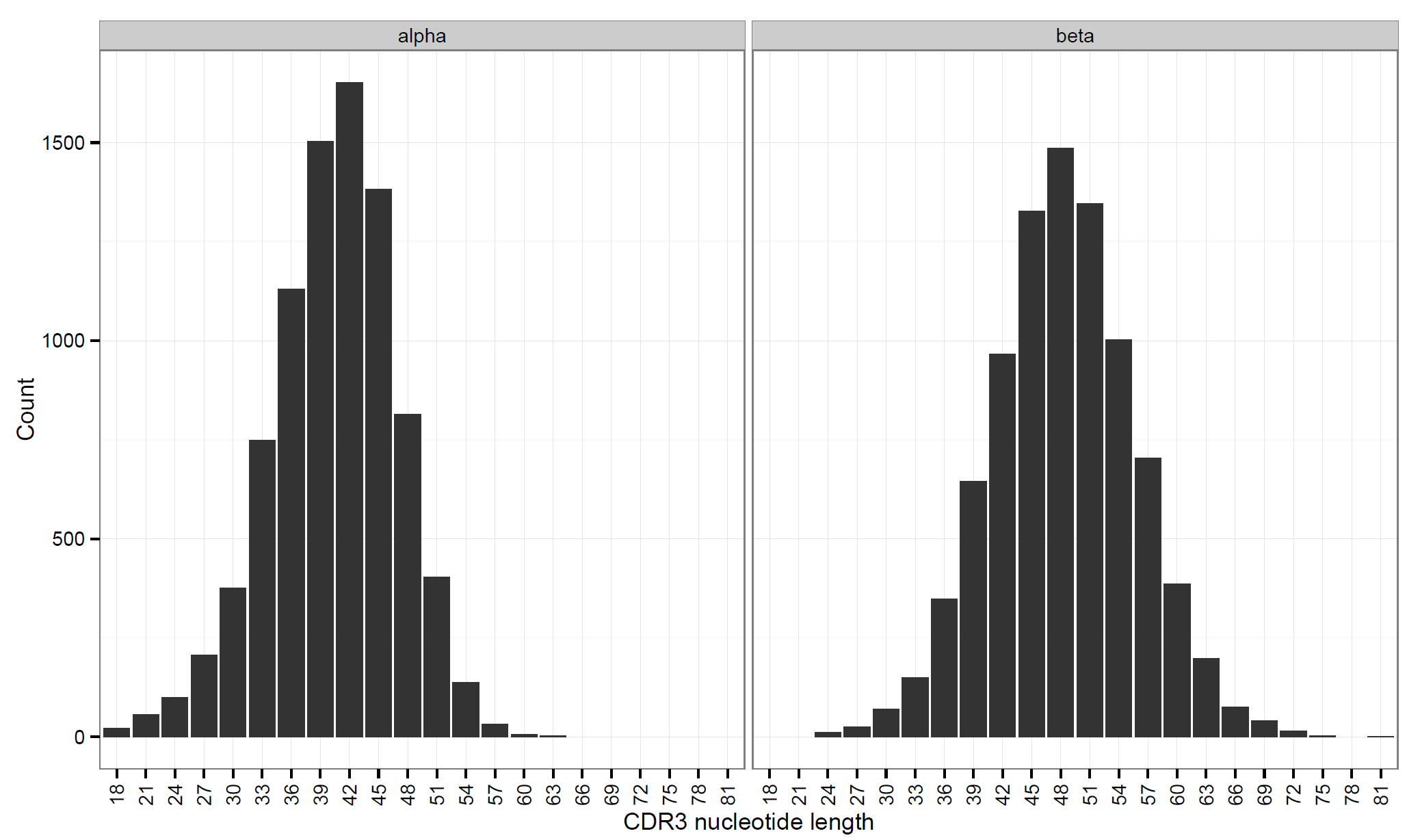
 **Supplementary Figure 1.** Length distributions of *in silico* generated CDR3 sequences. CDR3α and CDR3β sequence lengths plotted separately. CDR3α has mean 40.35 (standard deviation 6.54), and CDR3β has mean 48.29 (standard deviation 7.41).


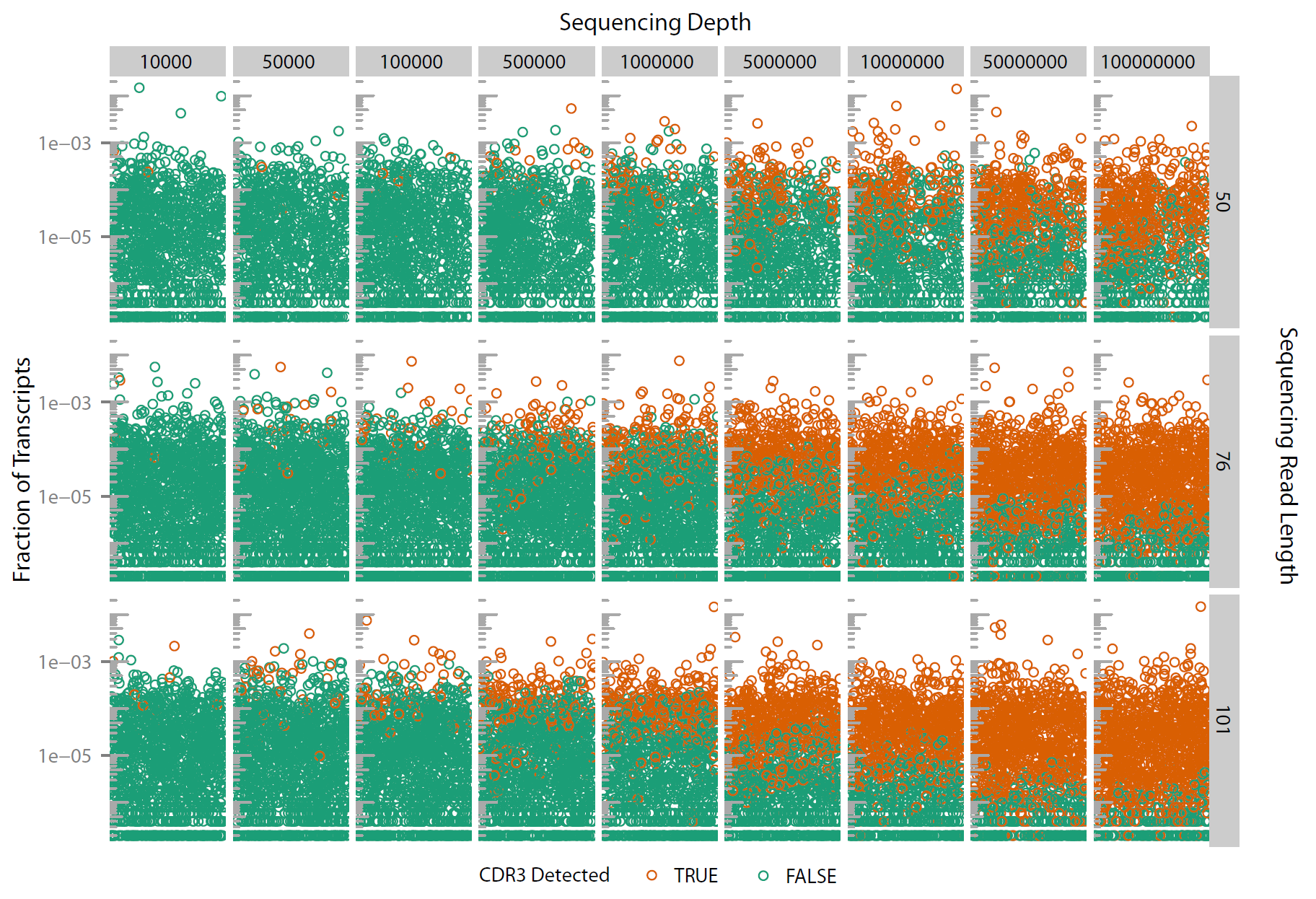


**Supplementary Figure 2.** TCR transcript abundance vs. sequencing depth. For the *in silico* data, the simulated TCR transcript abundance was tracked. Simulated RNA libraries which were sequenced deeper allowed lower abundance TCR transcripts to be detected.


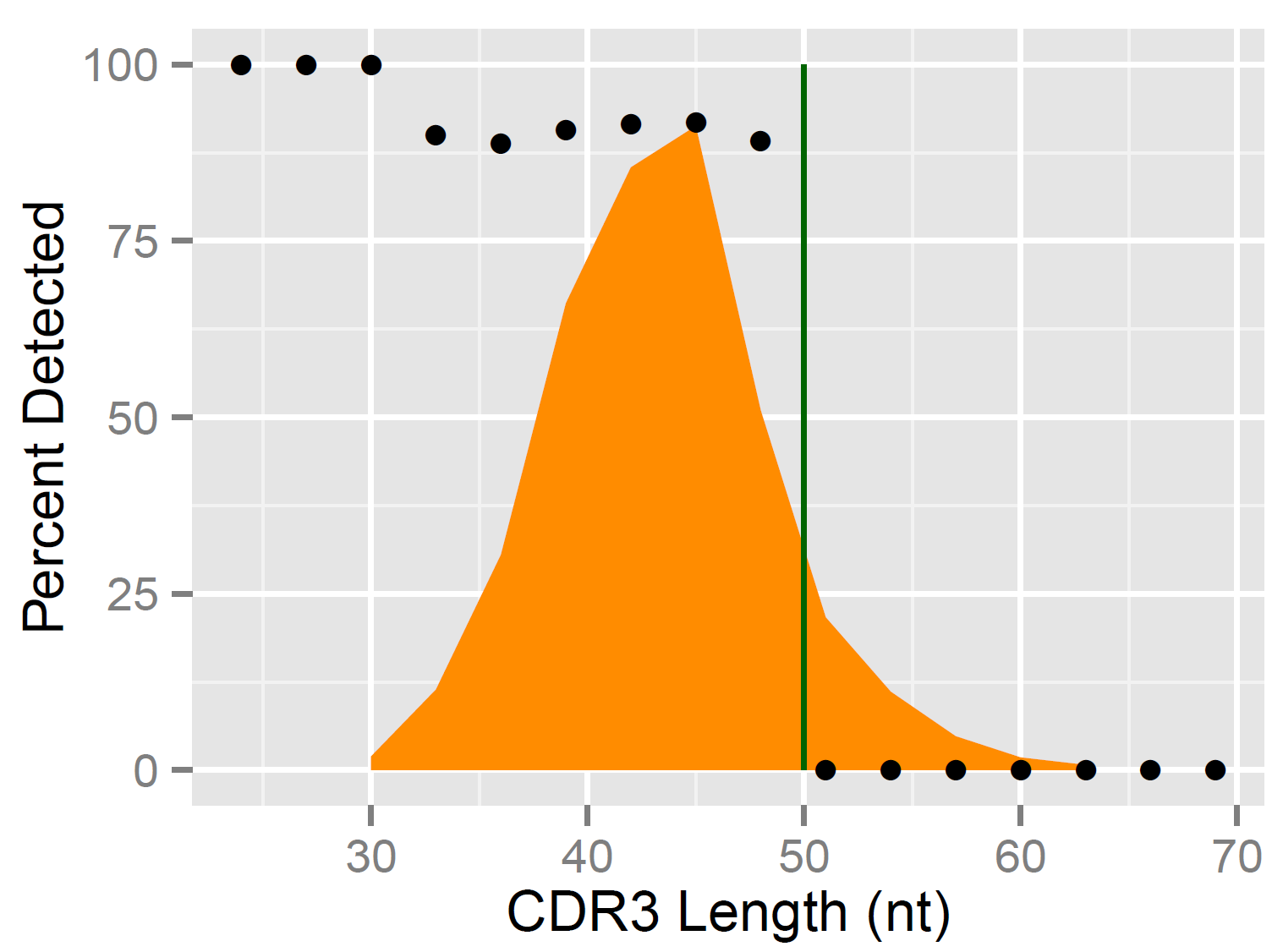


**Supplementary Figure 3.** Probability of detection of CDR3βs with varying lengths using error-free 50 nt reads centered on the CDR3 region. Orange density plot shows the distribution of CDR3β lengths in the normal population [2]. CDR3s which are longer than the read length (50 nts, green line) are not detected.


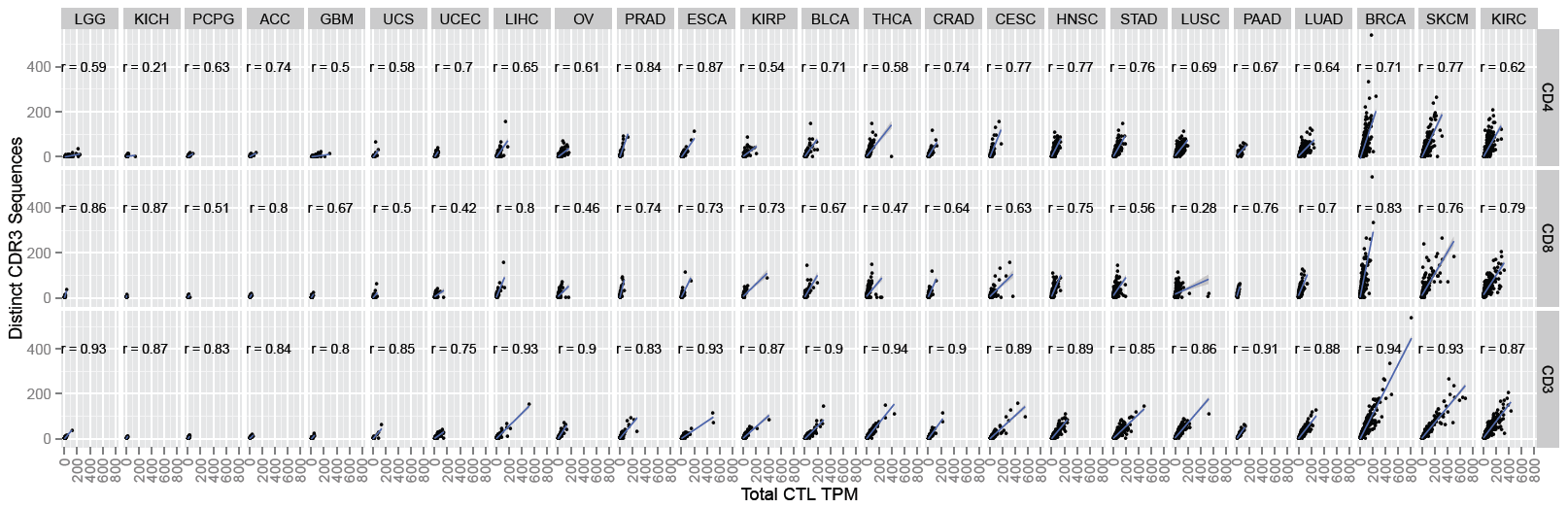


**Supplementary Figure 4.** Relationship between number of CDR3 amino acid sequences extracted and *CD4*, *CD8*, and *CD3* expression in tumor samples. Pearson correlation coefficients are displayed.


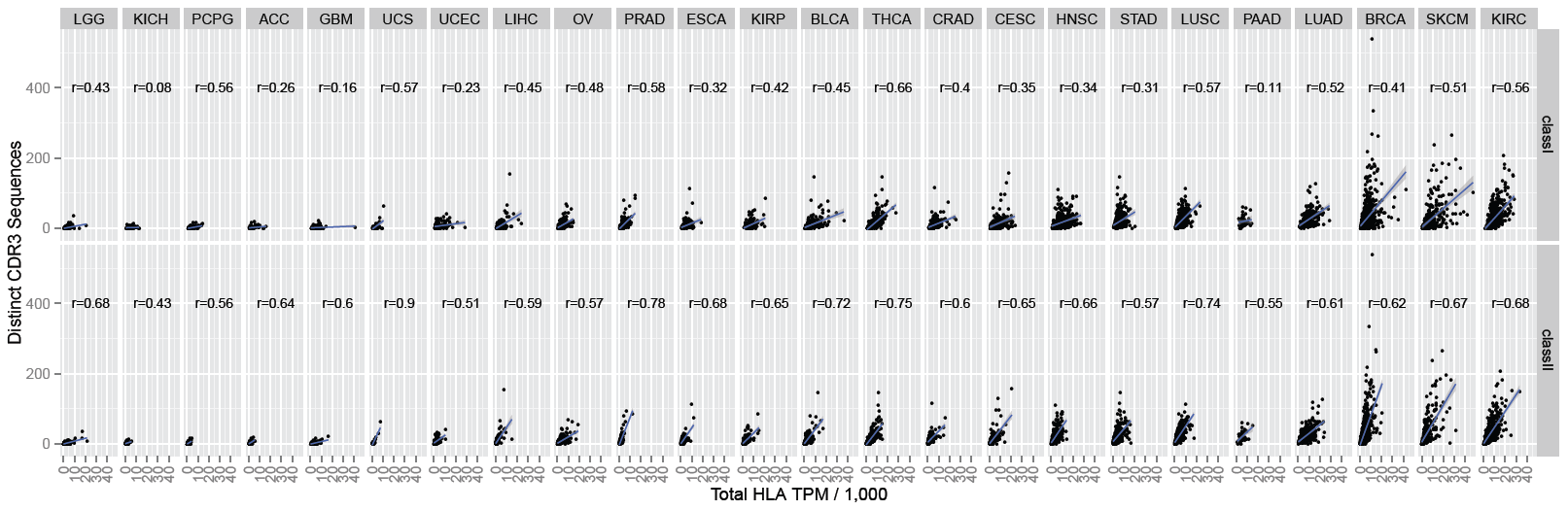


**Supplementary Figure 5.** Relationship between number of CDR3 amino acid sequences extracted and HLA Class I and Class II expression in tumor samples. Pearson correlation coefficients are displayed.


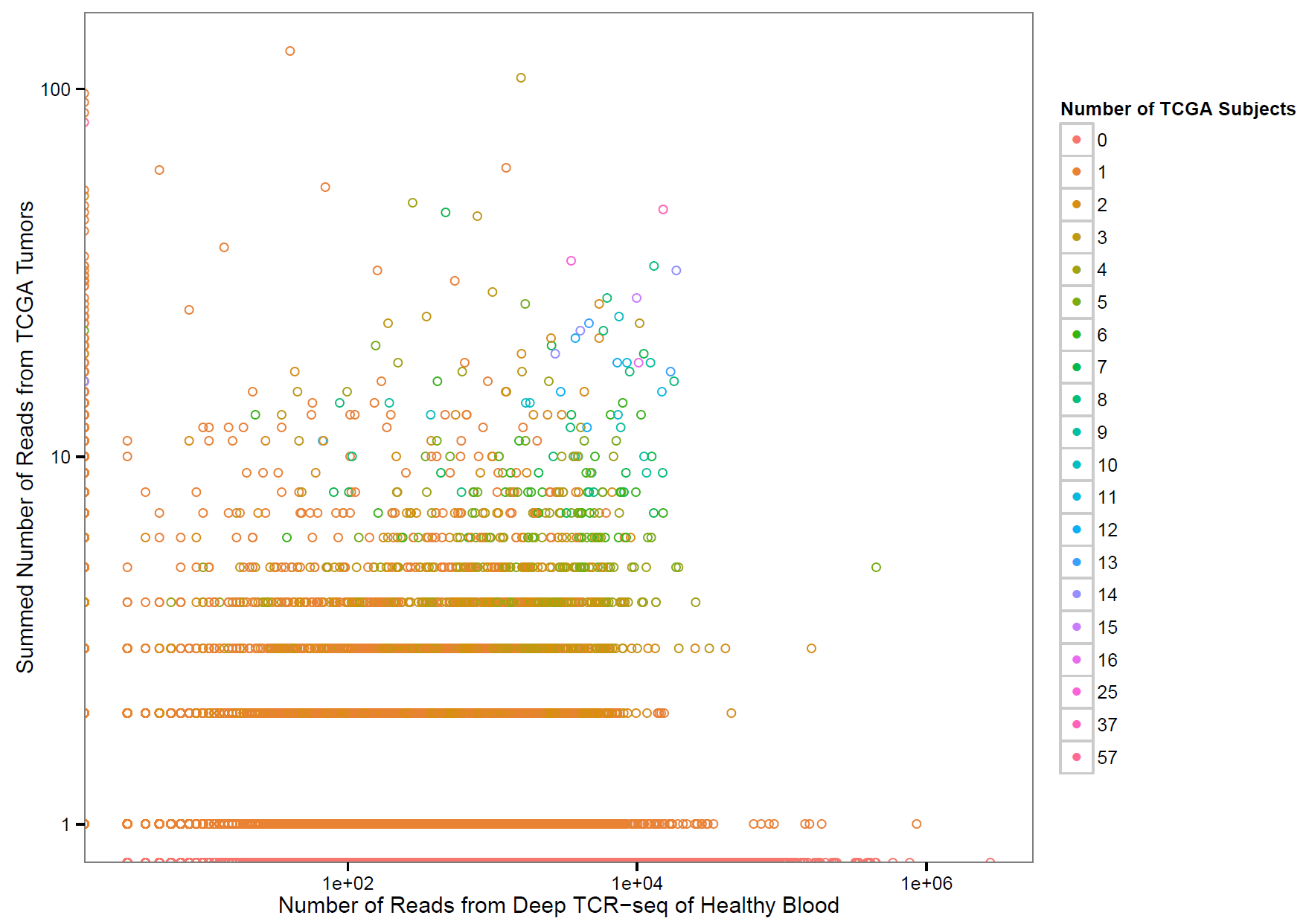


**Supplementary Figure 6.** Overlap between CDR3beta extracted from TCGA tumors and one individual’s deeply sequenced healthy blood sample. CDR3s that are found in multiple TCGA subjects are more likely to be found in the healthy individual’s TCR repertoire.
